# Transcriptome Analysis of Nodes and Buds from High and Low Tillering Switchgrass Inbred Lines

**DOI:** 10.1371/journal.pone.0083772

**Published:** 2013-12-30

**Authors:** Yixing Wang, Xin Zeng, Lila Peal, Yuhong Tang, Yanqi Wu, Ramamurthy Mahalingam

**Affiliations:** 1 Department of Biochemistry and Molecular Biology, Oklahoma State University, Stillwater, Oklahoma, United States of America; 2 Biology Department, Langston University, Langston, Oklahoma, United States of America; 3 Plant Biology Division, Samuel Roberts Noble Foundation, Ardmore, Oklahoma, United States of America; 4 Department of Plant and Soil Sciences, Oklahoma State University, Stillwater, Oklahoma, United States of America; University of Westminster, United Kingdom

## Abstract

In the last two decades switchgrass has received increasing attention as a promising bioenergy feedstock. Biomass is the principal trait for improvement in switchgrass breeding programs and tillering is an important component of biomass yield. Switchgrass inbred lines derived from a single parent showing vast variation in tiller number trait was used in this study. Axillary buds, which can develop into tillers, and node tissues, which give rise to axillary buds, were collected from high and low tillering inbred lines growing in field conditions. RNA from buds and nodes from the contrasting inbred lines were used for transcriptome profiling with switchgrass Affymetrix genechips. Nearly 7% of the probesets on the genechip exhibited significant differential expression in these lines. Real-time PCR analysis of 30 genes confirmed the differential expression patterns observed with genechips. Cluster analysis aided in identifying probesets unique to high or low tillering lines as well as those specific to buds or nodes of high tillering lines. Rice orthologs of the switchgrass genes were used for gene ontology (GO) analysis with AgriGO. Enrichment of genes associated with amino acid biosynthesis, lipid transport and vesicular transport were observed in low tillering lines. Enrichment of GOs for translation, RNA binding and gene expression in high tillering lines were indicative of active metabolism associated with rapid growth and development. Identification of different classes of transcription factor genes suggests that regulation of many genes determines the complex process of axillary bud initiation and development. Genes identified in this study will complement the current ongoing efforts in quantitative trait loci mapping of tillering in switchgrass.

## Introduction

Switchgrass is a C4 perennial grass that was selected in 1991 by the department of energy as a model herbaceous bioenergy crop for the development of renewable feed stock resource to produce transportation fuel [1]. Concerted efforts by several research groups have led to developing genetic and genomic resources to facilitate switchgrass breeding [2]–[6].

Biomass yield has been the principal trait for improvement in switchgrass breeding programs. Biomass yield is a complex trait controlled by a large number of genes, genotype and environmental factors [7]. In rice, it has been shown that final tiller number, girth, leaf length, individual tissue weights (leaves, sheaths, and stems), and days to maturity were positively correlated to final biomass [7], [8]. Using 11 lowland switchgrass populations tested in two locations, biomass yield was positively correlated with tiller number per plant with correlation coefficients of 0.60 to 0.68 [9]. Positive correlations between biomass yield and tillering ability, plant height, and stem thickness in switchgrass have been reported [10], [11]. Moderate overexpression of a rice miR156 precursor in switchgrass lead to 58%–101% more biomass yield compared with control plants [12]. Consistent with the earlier field studies, it was reported that the improvement in biomass yield was mainly because of the increase in tiller number [12]. Overall, these studies indicate that tiller number can be used as a key selection trait for switchgrass biomass improvement.

Tillering or branching is one of the most important agronomic traits that determine plant architecture and ultimately biomass. During this complex process, expression of many genes must be fine-tuned. Several studies have shown that transcription factors (TFs) play a key role in lateral meristem initiation and development. MYB transcription factor [13] and Lateral suppressor (Ls) gene [14] in tomato, and REVOLUTA (REV) gene [15] and a basic helix-loop-helix (bHLH) protein ROX [16] in *Arabidopsis,* are essential for formation of lateral meristems in dicots. Several TFs have also been reported as important regulators for vegetative branching in monocots. In maize, *teosinte branched 1* (*TB1*) controls major difference in plant and inflorescence architecture between cultivated maize and teosinte, its wild ancestor [17]–[20]. *BARREN STALK1* (*BA1*), a bHLH TF, and *BARREN STALK FASTIGIATE1* (*BAF1*), a TF containing an AT-hook DNA binding motif, are required for ear initiation in maize [21], [22]. *MONOCULM 1* (*MOC1*), a rice homolog of *Lateral suppressor* (*Ls*) in tomato [14], encoding a GRAS family transcription factor, has been determined as a key component that controls the formation of tiller buds and expressed mainly in the axillary buds [23]. *O. sativa* homeobox 1(OSH1) and TEOSINTE BRANCHED1 (TB1), have been proposed to act downstream of MOC1 in promoting rice tillering [8]. Rice *TB1* is an ortholog of the maize (*Zea mays*) *tb1* gene that is expressed in axillary meristems and regulates outgrowth of this tissue [24], [25]. *LAX PANICLE* (*LAX*), encoding a bHLH, an ortholog of maize *BA1* and *Arabidopsis REGULATOR OF AXILLARY MERISTEM FORMATION* (*ROX)*, and *SMALL PANICLE* were also reported as major regulators of axillary meristem formation in rice [26], [27]. These studies indicate that a gamut of TF encoding genes are involved in tillering trait in different plant species.

Several genetic maps for switchgrass have been published using the genomic simple sequence repeats (SSRs) [2], [28] and EST-SSR markers [29]. However, there are no reports of mapping quantitative traits in this plant. Advances in RNA sequencing technologies have led to detailed analysis of switchgrass transcriptomes [3], [5]. Recently, an Affymetrix genechip was designed based on 454 sequencing of more than 11.5 million ESTs. These genechips were used for compiling a switchgrass gene expression atlas comprised of several different tissue types and developmental stages collected from the popular cultivar Alamo [5].

In this study we examined transcriptomic differences in axillary buds and nodes in switchgrass lines derived from an inbred population that show extreme variation for tiller number trait. We chose to examine these two tissues since nodes give rise to buds, and buds under the right conditions can generate tillers. Identifying biomarkers associated with tillering trait will enable marker-assisted selection and accelerate the pace of developing high biomass yielding switchgrass cultivars.

## Materials and Methods

### Switchgrass Tissue Samples

**Figure 1:**
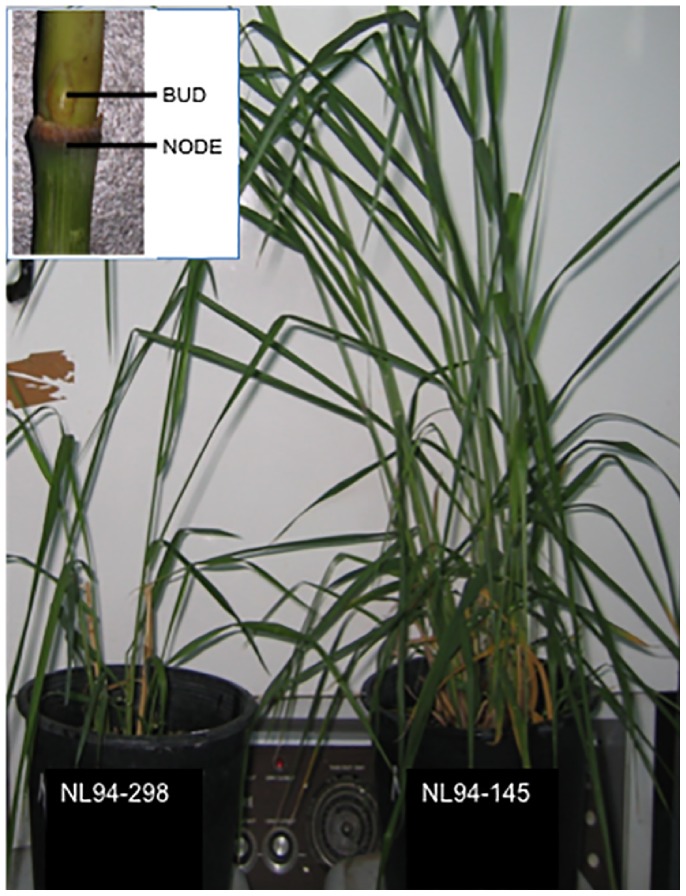
Variation in tillering trait of switchgrass lines used for this study. Low tillering (NL94-298) and high tillering line (NL94-145) from an inbred switchgrass population was selected. Inset shows magnified view of the biological material used for the affymetrix genechip analysis. The axillary buds and the non-green nodal region underneath were carefully excised out under a dissection scope.

Two first-generation switchgrass inbred lines derived from selfing a parental genotype ‘NL94 LYE 16×13’, which was selected from the Oklahoma State University northern lowland breeding population in 2007 [30], was used in this study. NL94/145 was tall, vigorous and high tillering. The NL94/298 inbred line was short and produced fewer tillers than NL94/145 (Figure 1). Several plants of these two lines were harvested from Agronomy field plots, Stillwater, OK. Stems of the high tillering line had 2–3 phytomers, while the low tillering line had 1–2 phytomers. Vegetative buds from first phytomer and node regions from second phytomer were cut out using a sharp scalpel under a dissection scope and immediately frozen in liquid nitrogen. Tissues from multiple plants of these two contrasting inbred lines growing in different sections of the field were pooled together to capture the biological variability.

### RNA Isolations

Total RNA from the four tissue samples (high tiller buds, high tiller nodes, low tiller buds and low tiller nodes) was isolated using Qiagen RNEasy Plant Isolation kit. Quality of the RNA was tested using BioAnalyzer (Agilent).

### Switchgrass Affymetrix Genechip Hybridizations

Purified RNA (500 ng) was amplified and labeled using the GeneChip 3′ IVT Express Kit (Affymetrix, Santa Clara, CA). Labeled RNA was hybridized to switchgrass Affymetrix genechips [6]. RNA from two independent preparations was used for replicated array hybridizations.

### Affymetrix Data Analysis

Data normalization was conducted by robust multi-array average [31]. Probesets with average signal intensity of more than 28 in replicate hybridizations in one or more tissues were selected for further analysis. Log_2_ transformed intensity values of the selected 8271 probesets were subjected to K-means clustering using Genesis software [32]. Using the normalized signal intensities, fold-change ratios between the high and low tillering lines were computed for the buds and node tissues. Probesets showing a greater than 2-fold ratio change were selected for further analysis. All the genechip datasets are available in MIAME-compliant format through ArrayExpress under the accession number E-MTAB-1899.

### Gene Ontology Analysis using AgriGO

Rice homologs for the selected switchgrass probesets were retrieved from switchgrassgenomics.noble.org and used for gene ontology (GO) analysis using AgriGO program [33]. Singular enrichment analysis was used to identify enriched GO terms in each of the sub-clusters specific for high and low tillering lines identified by K-means clustering described above. Parametric analysis of gene set enrichment (PAGE) tool was used to identify GO terms significantly associated with high tillering buds/nodes versus low tillering buds/nodes comparisons. PAGE method is based on Central Limit Theorem in statistics. It takes into account gene expression level and uses a two-tailed test to count Z score. The calculation of p-value was done using R software.

### Real-Time PCR Analysis

Plants of NL94/145 and NL94/298 were propagated in the growth-chamber using the axillary bud cuttings. Growth chamber was maintained with 16-hour/8-hour day/night periods and 26° C/20°C day/night temperatures. Total RNA was isolated from the buds and node tissues of these plants. One microgram of total RNA was used for cDNA synthesis. Gene specific primers were designed to amplify an approximately 100 base pair fragment of the target gene. Using a 1∶10 dilution of cDNA, the reactions were set up as follows: 5 µL Thermo Scientific Maxima SYBR Green/ROX qPCR Master Mix (2X), 1 µL Primer Mix (2 uM forward and reverse primers), 3 µL water, and 1 µL diluted cDNA. Quantitative PCR was run using the Applied Biosystems 7500 RealTime PCR System using the following parameters: initial denaturation at 95°C for 5 min, 40 cycles of denaturation at 95°C for 15 sec, and annealing/extension at 60°C for 60 sec. This was followed by a melting curve analysis. Fold-change in gene expression was calculated using the delta delta Ct (ddct) method. Amplification of the actin gene was used for data normalization.

## Results and Discussion

### Choice of Switchgrass Lines and Tissues used in This Study

Several field studies have indicated that tiller number trait is positively correlated with the biomass yield in switchgrass plants [9], [11], [34]. Thus, identifying the genes crucial for tillering will provide a valuable resource for manipulating this important trait and improving switchgrass biomass production. However, being a polyploid and outcrossing species, switchgrass presents formidable challenges for conducting genetic studies to identify loci associated with economically important traits such as tillering. An inbred switchgrass population was developed by selfing a heterozygous parent of a northern lowland genotype, NL 94 [2]. Progeny from this inbred population showed remarkable variability for the tiller number trait (Figure 1). Since all genetic variability is attributable to a single parent, the low and high tillering lines selected from this population provided a simplified genetic background as compared to a population derived from crossing. Bulking the tissue samples from several independent plants further aided in homogenizing the genetic background.

Switchgrass axillary buds on the nodal junctions when cultured on the MS media or under appropriate soil moisture conditions can generate tillers in a span of one week (File S1). In fact, the most effective method of micro-propagation for clonal replicates for switchgrass seed production is from the axillary buds of the lower nodes [35], [36]. Based on these observations we surmised that transcriptomic differences in the axillary buds of these contrasting inbred lines would be a valuable resource for identifying genes involved in tillering. Since the buds arise from nodal regions of the tillers, we also examined the differences in the nodal transcriptome of these two inbred lines.

Multiple plants of NL94/298 and NL94/145 genotypes growing in swards were harvested to collect a sufficient amount of bud and node tissues for RNA isolations. This sampling procedure contributed towards capturing the biological variability within the samples (low or high tillering), despite the fact all sampling was conducted during a single growing season. Two independent RNA isolations were undertaken for conducting microarray hybridizations. Switchgrass Affymetrix array containing 122,868 probesets corresponding to 110,208 unigene transcripts [6] was used. Correlation between the two replicate hybridization experiments with bud and node tissues was 0.98 and 0.97, respectively. These values indicate the high technical reproducibility of the probe preparation, labeling, and genechip hybridization procedures.

### Clustering and Gene Ontology Enrichment Analysis

**Figure 2:**
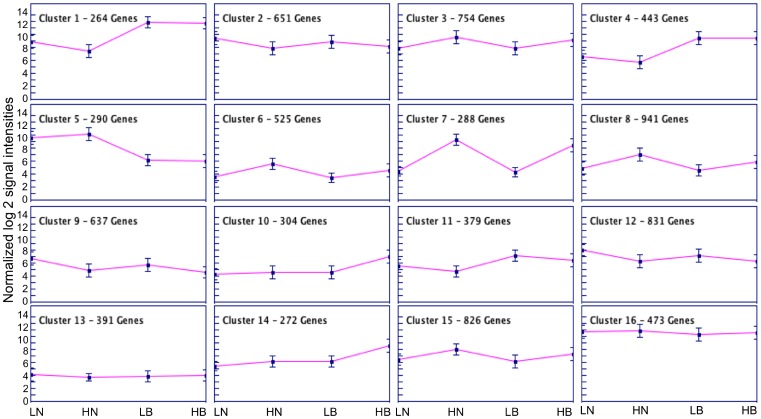
Cluster analysis using signal intensities from Affymetrix genechip hybridizations. K-means cluster analysis using the normalized average log 2 signal intensities from buds and nodes of high and low tillering lines was conducted using the GENESIS program. HN: High tillering nodes; HB: High tillering buds; LN: Low tillering nodes; LB: Low tillering buds. K = 16 clusters and 100 iterations.

We tested the null hypothesis that gene expression patterns are unique for each genotype (low and high tillering line) and for each tissue sample used in this study (nodes and buds). For this analysis the normalized filtered hybridization signal intensities were used. Based on the signal to noise ratio from these experiments and other switchgrass genechip experiments conducted at Noble Foundation, a normalized transcript level of 28 was set as a threshold, below which a gene was determined as not expressed. Approximately 7% of the probe sets were selected based on this cut-off criterion (8269 out of 122,868) (Files S2–S5). K-means cluster analysis with K = 16 revealed six major patterns of gene expression (Table 1, Figure 2). Five clusters (3, 6, 7, 8, and 15) contained probesets with higher expression in the buds and nodes of high tillering line and accounted for 40% of the entire set. Three clusters (2, 9, 12) contained probesets with higher expression in the buds and nodes of the low tillering line. Two clusters (13, 16) contained probesets that were expressed similarly in the two genotypes and in the two tissue types. Three clusters (1, 4, and 11) contained probesets that were more strongly expressed in the bud tissues than in the nodes. The opposite pattern was observed in cluster 5, wherein probesets showed higher expression in nodes compared to buds. Interestingly, two clusters (10, 14) contained probesets that exhibited higher expression in the bud tissues of only the high tillering line.

**Table 1:** Summary of K-means clustering and AgriGO analysis.

Clusters^a^	# of probesets^b^	With rice ids^c^	RiceIDs with GOs^d^	# of significant GOs^e^
2,9,12 (low buds and nodes)	2119	1497	1039	49
3,6,7,8,15 (high buds and nodes)	3324	2501	1484	58
1,4,11 (buds)	1086	942	719	85
10,14 (high tillering buds)	576	458	369	63
5 (nodes)	290	244	184	0
13,16 (all samples)	864	663	550	40

^a^ Clusters numbers are related to figure 2.

^b^ The total number of probesets showing similar expression patterns based on figure 3.

^c^ Number of unique rice gene identifiers associated with switchgrass probesets.

^d^ Rice identifiers for which GOs were retrieved from the MSU rice genome annotation project (http://rice.plantbiology.msu.edu/downloads_gad.shtml).

^e^ Number of enriched GO terms associated with sub clusters based on singular enrichment analysis in AgriGO (Files S6–S8).

In order to examine the nature of the genes in these clusters, the closest rice orthologs for the switchgrass probesets were retrieved from switchgrassgenomics.noble.org. Of the 8269 probesets selected based on the intensity of expression, rice identifiers were retrieved for 6304. The nearly 25% drop in number of rice loci may be due to grouping of gene family members of the allopolyploid switchgrass to single rice orthologs (Table 1). This has been well documented in recent studies of the lignin and C4 photosynthesis pathway genes wherein multiple switchgrass orthologs were identified for each rice gene [37], [38].

**Figure 3:**
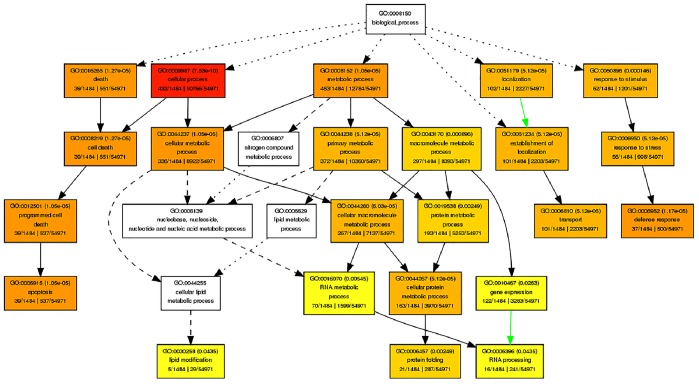
Enriched gene ontologies in differentially expressed genes of high tillering switchgrass inbred line. Singular enrichment analysis was performed in AgriGO to identify enriched gene ontologies associated with high tillering lines. Each box shows the GO term number, the p-value in parenthesis, and GO term. First pair of numerals represents number of genes in input list associated with that GO term and number of genes in input list. Second pair of numerals represents number of genes associated with a particular GO term in the rice database and total number of rice genes with GO annotations in the rice database. Box colors indicates levels of statistical significance: yellow = 0.05; orange = e-05; and red = e-09.

In order to comprehend the unique aspects of nodes and buds with reference to their transcriptomes, the orthologous rice genes were subjected to gene ontology (GO) enrichment analysis using the AgriGo program [33]. GOs for approximately 70% of the rice genes were retrieved using the MSU 6.0 version annotations in AgriGO. Using the singular enrichment analysis in AgriGO the enriched GO terms were identified for each of the six unique expression clusters described above. In the biological process category, gene expression, lipid modification, protein folding, and RNA processing were unique terms associated with high tillering line (Figure 3). Diacyclic graphs showing the enrichment of GOs associated with biological process category for other clusters are in supplementary figures (Files S6–S8).

Majority of the genes in the gene expression category were transcription factors (TFs). Five different WRKY TFs (WRKY3, 13, 42, 46, 74), four AP2 domain containing proteins, basic zipper (bZIP) TFs, B3 DNA binding domain containing proteins, and no apical meristem (NAM) proteins were strongly expressed in high tillering line. Though most of the studies of WRKY TFs are associated with stress/defense responses, several of them are involved in development [39]–[41]. WRKY13 has been shown to regulate WRKY42 and WRK74 in response to pathogen infections in rice [42]. The role of these WRKY TFs in tillering merits further attention. Auxin response factors, HSFs, MADS box factors (MADS13, 75) homeobox leucine zipper, NAC domain containing proteins, and ethylene responsive Aintegumenta are other TFs that were up regulated in the high tillering line when compared with their expression in the low tillering line. Aintegumenta (Ant) regulates cell proliferation and organ growth by maintaining meristematic competence of cells during organogenesis [43] and can promote initiation and growth of lateral organ primordia [44]. It is tempting to speculate that enhanced expression of Ant-like genes in the high tillering switchgrass may play a crucial role in promoting this trait.

Several genes associated with general transcriptional machinery were differentially expressed between the high and low tillering lines including transcription initiation factor IIf beta subunit, transcription elongation factor SPT5 homolog, elongation factor 1 gamma subunit, mRNA splicing factor, and several RNA polymerase subunits. In rice, disruption of a transcription elongation factor, TEF1, led to reduced tillering and also affected stress response gene expression [45]. We speculate that some of the genes associated with basal transcriptional machinery may be important players in coordinating shoot branching in switchgrass.

Chromatin remodeling processes play a central role in establishing transcriptional programs required for organ initiation and differentiation. For the sequential production of lateral organs, meristem genes should be stably repressed outside the meristematic regions and differentiation genes should be stably expressed in developing tissues [46]. Several chromatin remodeling proteins such as BROMO adjacent homology (BAH) domain containing protein, histone acetyl transferase (HAC1), High Mobility Group (HMG1/2), and cytosine specific DNA methylase were up-regulated in the high tillering line and merit further attention.

One of the interesting observations was the enrichment of the GO for small molecule biosynthesis in the low tillering line. Among the genes in this GO, many were involved in biosynthesis of amino acids. In Arabidopsis it was shown that an increase in the seed lysine amino acid pool led to autotrophic growth retardation, affecting seedling establishment [47]. In potato plants, increasing the levels of methionine led to an overall increase in the amino acid pool and more importantly led to growth retardation [48]. Based on the above studies we speculate that the increased transcription of amino acid biosynthesis genes could be a deterrant for tillering.

Increased expression of glutathione (GSH) biosynthesis genes in the low tillering line is intriguing. GSH is an important player in cell redox homeostasis that in turn impacts the cell cycle regulation in plants [49]. Interestingly, higher levels of oxidized glutathione (GSSG) improved shoot meristem structure and promoted embryo maturation [50]. We speculate the higher expression of GSH biosynthetic genes could lead to higher levels of GSH and this in turn can inhibit tillering. Analysis of the GSH and GSSG levels in the high and low tillering plants will render further credence to this hypothesis.

### Comparative Analysis of Gene Expression in High and Low Tillering Lines

**Figure 4:**
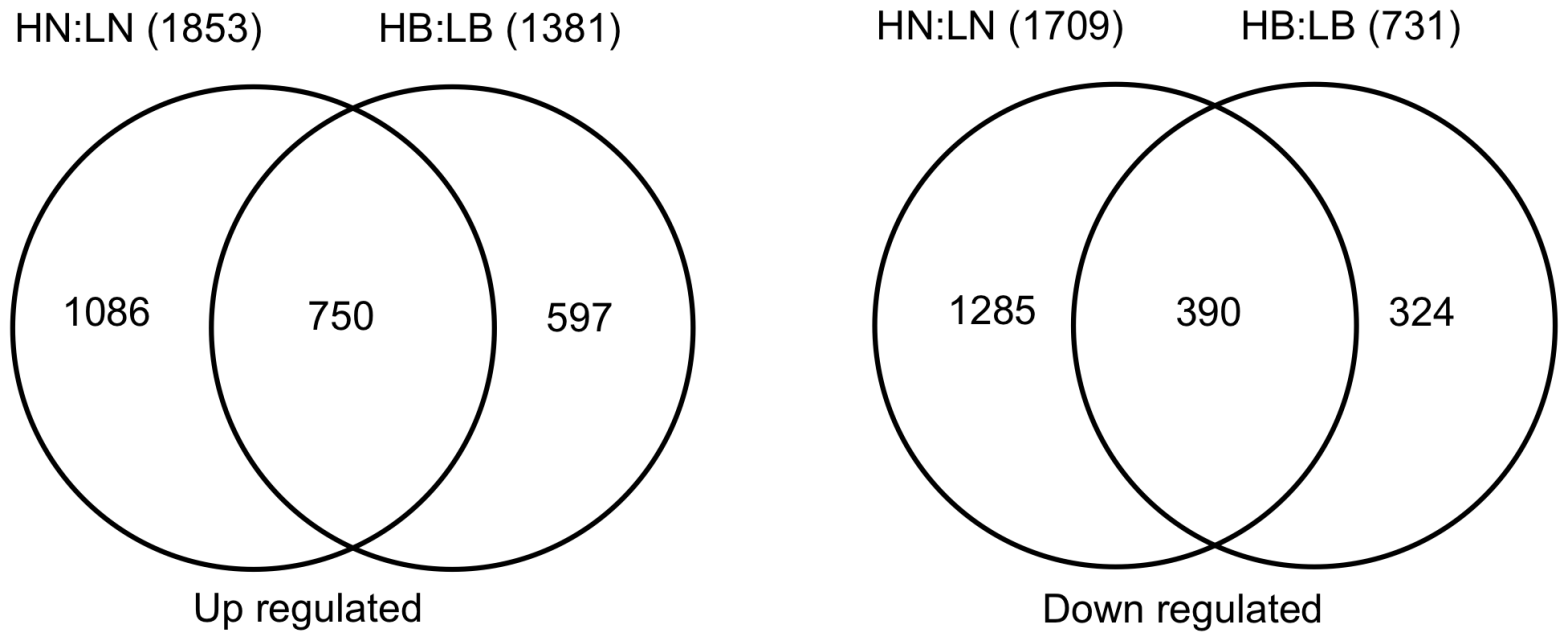
Venn diagram of differentially expressed genes in switchgrass inbred lines. The venn diagram shows the number of differentially expressed genes in nodes and buds of the high and low tillering lines. Up and down regulated genes are shown separately. HN:LN represents genes differentially expressed in high tillering nodes compared with low tillering nodes. HB:LB represents genes differentially expressed in high tillering buds compared with low tillering buds.

Differential expression of genes between the high and low tillering lines was computed separately for buds and nodes using average normalized signal intensities. Ratios that were greater than 2-fold or less than 0.5 and with p-values <0.05 were considered as differentially expressed in this analysis. The comparative transcriptome analysis was undertaken for only those switchgrass probesets that had a rice ortholog. Given the fact that the bud tissues are metabolically more active compared to the node tissues we anticipated larger number of genes to be differentially expressed in the former. On the contrary, the total number of differentially expressed probesets was nearly 1.7 times more in the nodes (3562) than in the buds (2112) (Figure 4). Interestingly, the nodes of the low tillering lines had larger numbers of unique probesets with higher expression compared with the nodes of the high tillering line. However, in the bud tissues, the high tillering line had approximately 50% more probesets that were differentially expressed compared with the low tillering line. A small fraction of the probesets showed opposite patterns of expression in the buds and node tissues in both high and the low tillering lines. These data suggested that even though the buds and nodes are so closely juxtaposed, their transcriptome profiles were unique. Further, these differences were more pronounced between the high and the low tillering lines.

**Figure 5:**
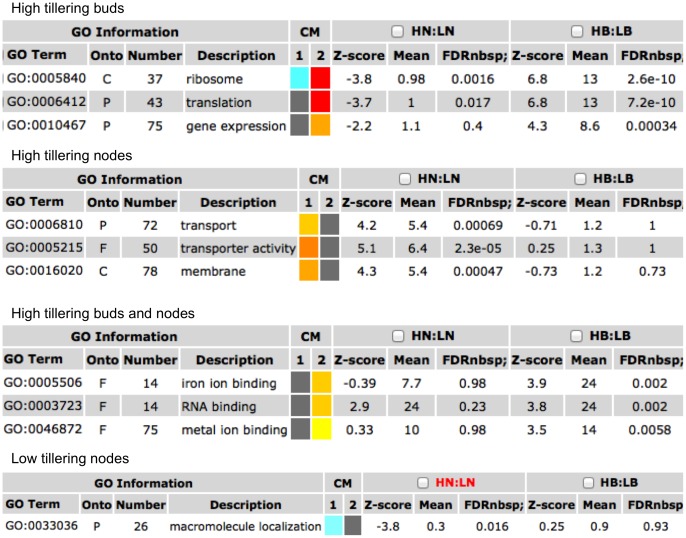
Parametric analysis of gene expression and gene ontologies in switchgrass inbred lines. Only significant GO terms associated with the comparisons of high and low tillering lines were selected for display. CM refers to colorful model. Box colors indicates levels of statistical significance: yellow = 0.05; orange = e-05; and red = e-09. Different shades of blue color indicate the extent of down regulation. Onto refers to the ontology category: C- Cellular component; F- Molecular function; P – Biological process. Numbers refer to the number of genes associated with the GO term. FDR refers to false discovery rates.

**Figure 6:**
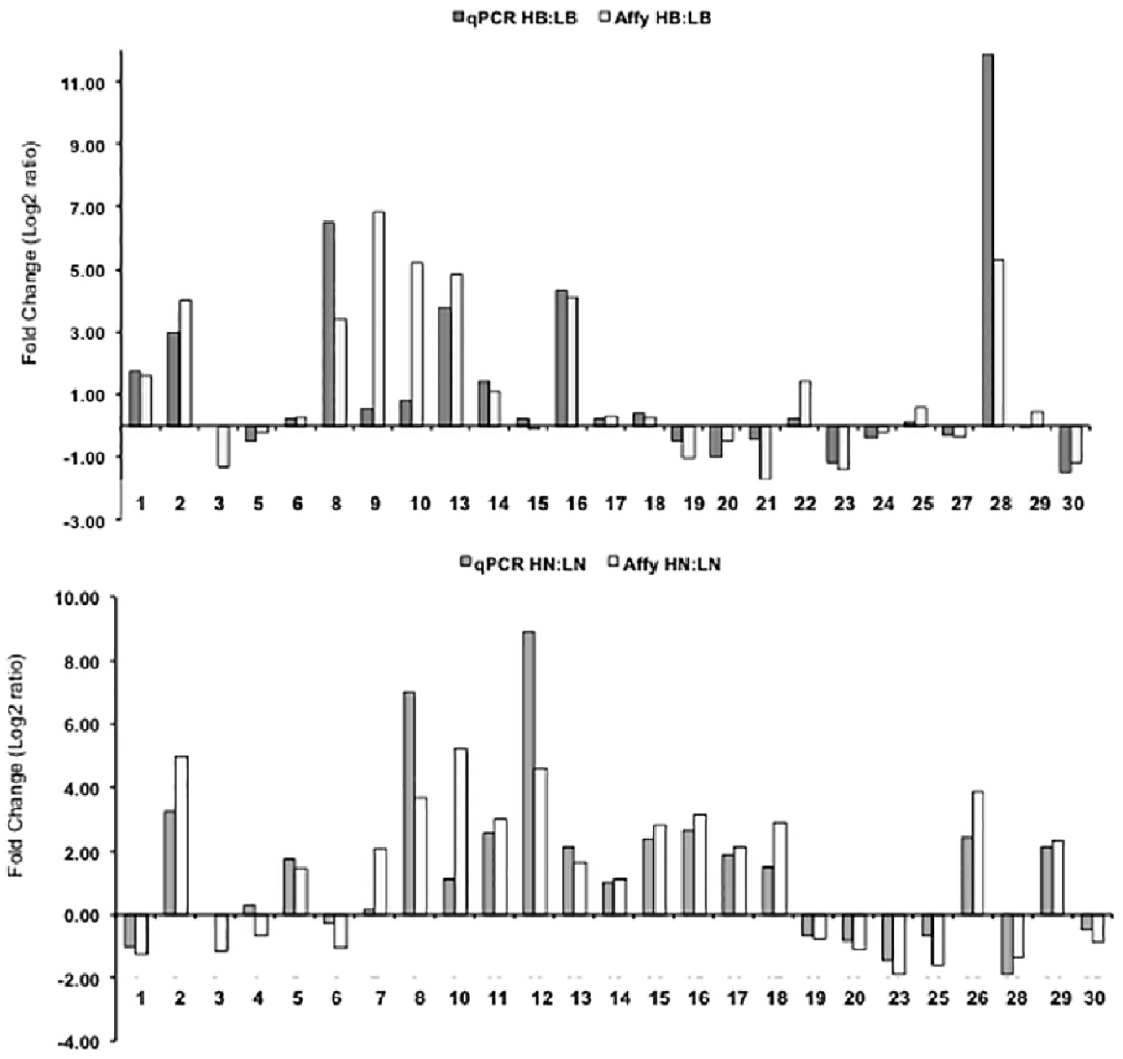
Comparing qPCR and affymetrix genechip based fold-changes of gene expression in switchgrass. Quantitative PCR of select genes identified in the microarray analysis of high and low tillering switchgrass lines. HB:LB represents genes differentially expressed in high tillering buds compared to low tillering buds. HN:LN represents genes differentially expressed in high tillering nodes compared to low tillering nodes. Annotations of genes labeled 1–30 on the x-axis and the primers used for qPCR analysis are provided in File S9.

Parametric analysis of gene set enrichment provided some interesting insights into the differences between the high and low tillering lines (Figure 5). Among the genes that were up regulated in the buds of high tillering line, GOs for translation and gene expression were highly significant and most of the genes in the former group were ribosomal proteins. This indicates that the bud tissues are metabolically very active and the cells are committing a significant amount of resources on translational machinery to cater to the demands of robust growth observed in the high tillering line. In the gene expression category, a large number of transcription factors were identified and discussed above in detail. Expression of several TF genes was tested by qRT-PCR (Figure 6, File S9) using RNA from low and high tillering plants that were raised in the growth chamber. The selection of these genes was based on fold change ratios, annotations, and whether it was identified in the high or low tillering lines, buds or node tissues, or was common to both tissue samples. Only one primer pair (TF3) failed to give any amplification. Of the 24 genes with positive results in the bud tissues, 21 genes exhibited fold-changes comparable to the genechip data (Figure 6). Similarly, among the 24 genes with positive amplifications with node tissues, 20 genes exhibited fold-change ratios comparable to the array data (Figure 6). This independent confirmation of differential expression using qPCR further renders support to the validity of the genchip experiments.

In the nodes of the high tillering line, GO for transporter activity was significant. Elements absorbed from roots are redirected to nodes through the vascular bundles linked with roots, leaves and panicles. Transport of metals such as zinc and manganese are essential for normal growth of plants. Potassium, chloride, sucrose, copper, ascorbate, calcium, and metal cation transporters were up regulated in the nodes of high tillering lines. One chloride channel transporter is localized to the vacuole (Os12g25200) based on GO annotations. Chloride channel proteins localized to vacuoles are involved in nitrate homeostasis [51]. It is tempting to speculate that this chloride channel protein may be involved in nitrate transport in switchgrass high tillering lines. Four different peptide transporter genes were up regulated in the high tillering line. In rice it was recently shown that overexpression of a peptide transporter led to increased ammonium assimilation, prolific root growth and higher yields [52]. Two different Multi Antimicrobial Extrusion (MATE) efflux family proteins were identified in the high tillering line. In *Arabidopsis*, overexpression of the ZRZ gene, a MATE type transporter protein, led to faster production of leaves and enhanced growth of axillary buds [53]. Though most of the MATE family proteins are involved in detoxification, aluminum tolerance, and anthocyanin accumulation, the identification of ZRZ gene in axillary bud growth reveals a novel function for these proteins. In the wake of these studies, switchgrass MATE family proteins expressed strongly in the high tillering line warrant further attention.

GOs for iron ion binding and RNA binding were significantly enriched in both the nodes and buds of high tillering lines. Six different cytochrome P450 genes were identified. It is well known that cytochrome P450 gene family members are important for the biosynthesis of several phytohormones including giberellic acid, cytokinins, brassinosteroids and strigolactones, and that these second messengers are involved in the shoot branching pathway [54], [55]. RNA binding proteins (RBPs) with different RNA binding motifs, such as the RNA Recognition motif (RRM), la domain, and pumilio, was upregulated in the high tillering line. RBPs such as FCA and FPA are important for floral development [56], [57]. However, no RBPs have been shown to be involved in shoot proliferation and these candidate genes need further analysis to determine their precise role in tillering.

Among the genes up regulated in the nodes of the low tillering line, GO for macromolecule localization was enriched. In this GO category, 10 genes annotated as lipid transfer like proteins (LTPs) were identified. Most of the LTPs reported in various plant species are involved in pathogen defense responses and in fact these proteins were classified as a class of pathogenesis related proteins (PR-14) [58]. A carrot LTP protein was reported to be important for somatic embryogenesis [59] and in *Arabidopsis* reduction in the levels of AtLTP3 and AtLTP4 led to developmental defects such as dwarfed plants [60]. LTP1 epitopes were predominantly identified in the embryogenic cells of *Arabidopsis* while their presence was not detected in meristematic cells, suggesting the presence of these proteins as markers for change in cell fate [61]. A thorough analysis of the LTP-like proteins identified in switchgrass could reveal novel functions of this class of proteins associated with tillering trait.

Six different ras-related proteins and several proteins associated with vesicular transport including sec61, v-SNARE, importin, coatomer subunits, signal recognition particle, and syntaxin were identified in the nodes of the low tillering lines. Vesicular transport has been shown to be very important for auxin signaling [62]–[64] and in cytokinesis [65]. Changes in trafficking machinery impede cytokinesis, responses to hormones, and development [66]. Observed changes in expression of vesicular transport genes in conjunction with the LTPs in the nodal tissues of low tillering line may alter auxin homeostasis that in turn could retard branching.

### Prospects for Mapping and/or Cloning QTLs for Tillering in Switchgrass

Transcriptome analysis in the nodes and bud tissues of switchgrass inbred lines provided a glimpse at the gamut of molecular processes that could contribute to the phenotypic differences in tillering in these two genotypes. The enrichment of ribosomal proteins in the high tillering lines strongly supports higher vigor in these plants that favors rapid growth and development. The variety of specific transcription factors, RNA binding proteins differentially expressed in the two genotypes, can in turn impact a spectrum of transcriptional and post-transcriptional events associated with development. Enrichment of genes associated with iron binding that included a variety of cytochrome P450s suggests a role for the phytohoromone pathways in regulating tillering in switchgrass. Enrichment of genes associated with vesicle formation and lipid transport in low tillering lines implies the secretory pathway may have a hitherto unknown role in impeding shoot development.

Tillering is a quantitative trait in which several genes from multiple pathways must be finely tuned. Our transcriptome study using inbred lines with contrasting tillering habits confirms this in switchgrass. QTL mapping in switchgrass for identifying tillering associated genomic regions using this inbred population is currently underway. The differentially expressed genes, especially regulatory factors and signaling genes identified in this study, will be primary candidates for pursuing map-based cloning of QTLs associated with tillering trait.

## Supporting Information

File S1 **Tissue culturing of switchgrass nodal buds.** Surface sterilized phytomers containing a nodal bud flanked by an inch of stem on either end was placed in MS media for five days. Photographs were taken using a canon digital camera on the sixth day.
(TIF)

File S2 **List of probesets up regulated in buds of high tillering line.**
(XLSX)

File S3 **List of probesets up regulated in buds of low tillering line.**
(XLSX)

File S4 **List of probesets up regulated in nodes of high tillering line.**
(XLSX)

File S5 **List of probesets up regulated in nodes of low tillering line.**
(XLSX)

File S6 **Singular enrichment analysis using AgriGO to identify enriched gene ontologies associated with buds.**
(TIF)

File S7 **Singular enrichment analysis using AgriGO to identify enriched gene ontologies associated with buds and nodes of low tillering lines.**
(TIF)

File S8 **Singular enrichment analysis using AgriGO to identify enriched gene ontologies associated with buds of high tillering lines.**
(TIF)

File S9 **Annotations, gene expression and fold-change ratios of the genes selected for qPCR analysis.** Primer sequences used for the real-time PCR assays are listed.
(XLSX)
