# Supplementary figures and images for: Transcriptome Analysis of Nodes and Buds from High and Low Tillering Switchgrass Inbred Lines
Source: PLoS One. 2013 Dec 30;8(12):e83772. doi: 10.1371/journal.pone.0083772 (PMC3875486; doi:10.1371/journal.pone.0083772)

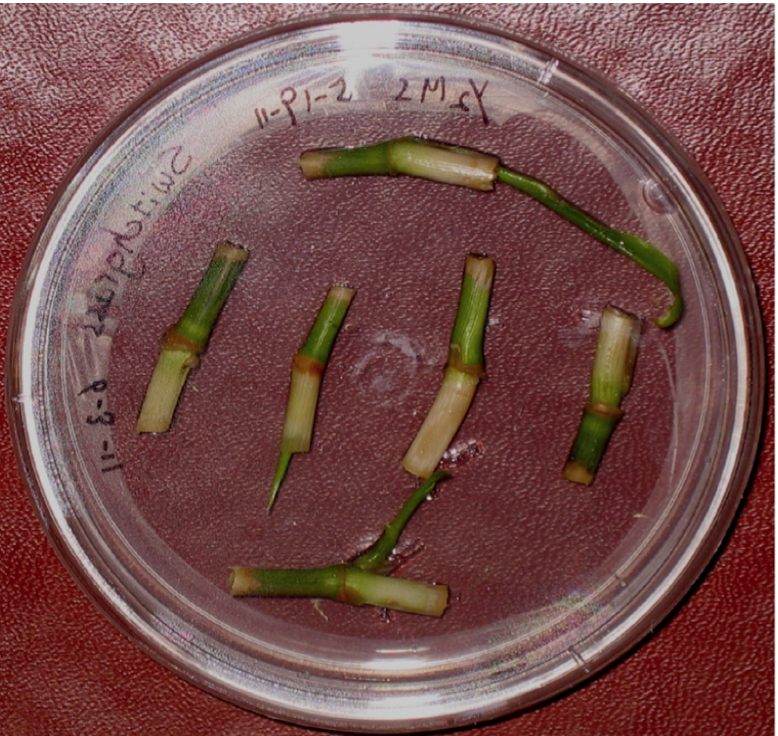

Supplement: File S1 — Tissue culturing of switchgrass nodal buds. Surface sterilized phytomers containing a nodal bud flanked by an inch of stem on either end was placed in MS media for five days. Photographs were taken using a canon digital camera on the sixth day. (TIF) [file pone.0083772.s001.tif]

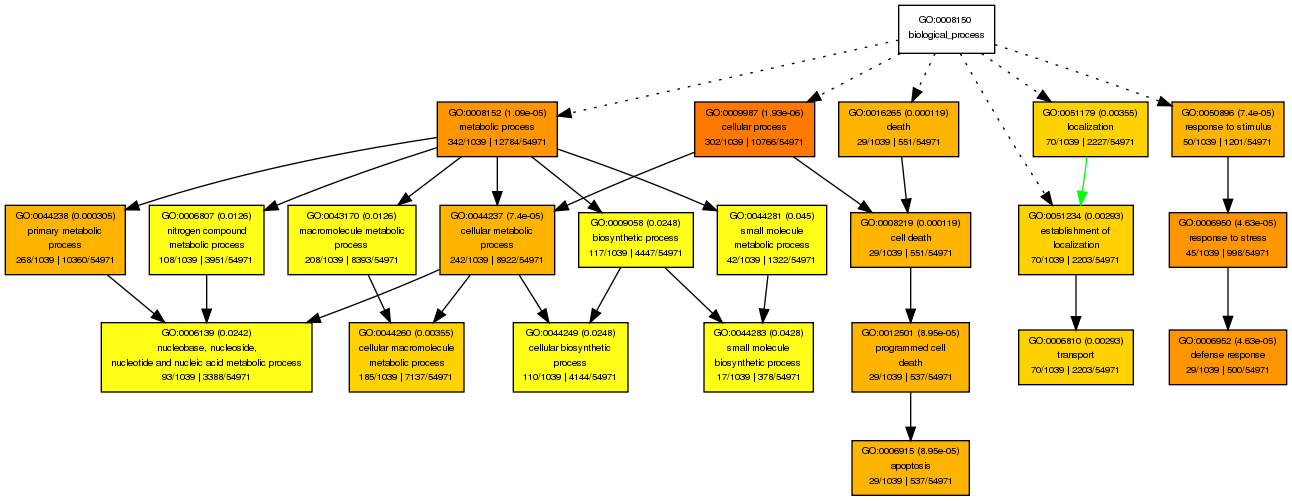

Supplement: File S6 — Singular enrichment analysis using AgriGO to identify enriched gene ontologies associated with buds. (TIF) [file pone.0083772.s006.tif]

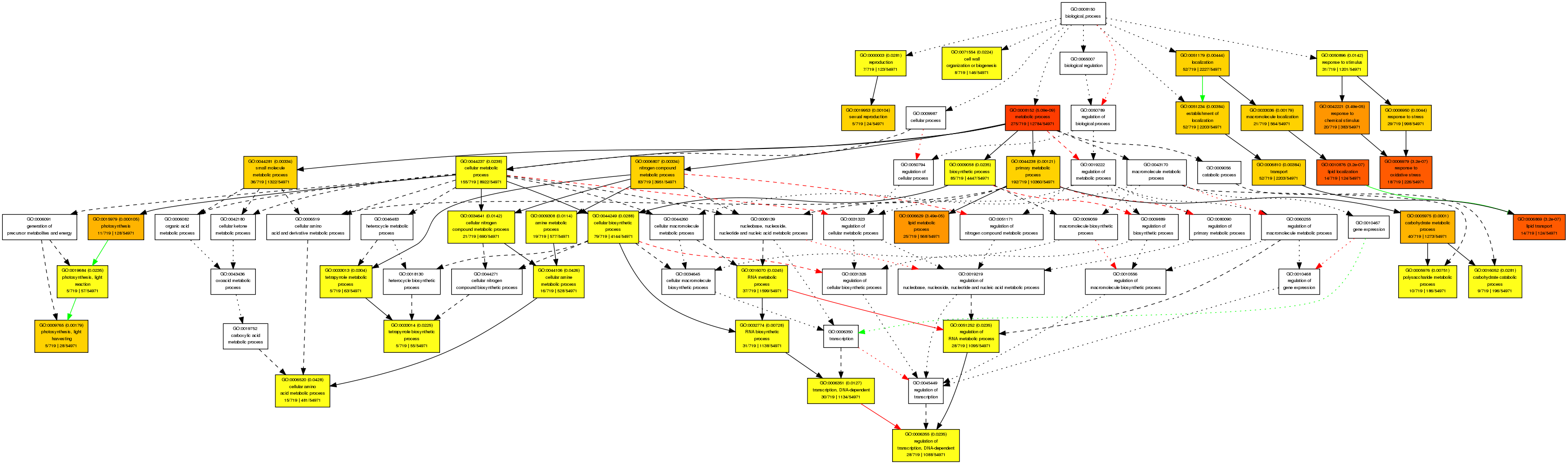

Supplement: File S7 — Singular enrichment analysis using AgriGO to identify enriched gene ontologies associated with buds and nodes of low tillering lines. (TIF) [file pone.0083772.s007.tif]

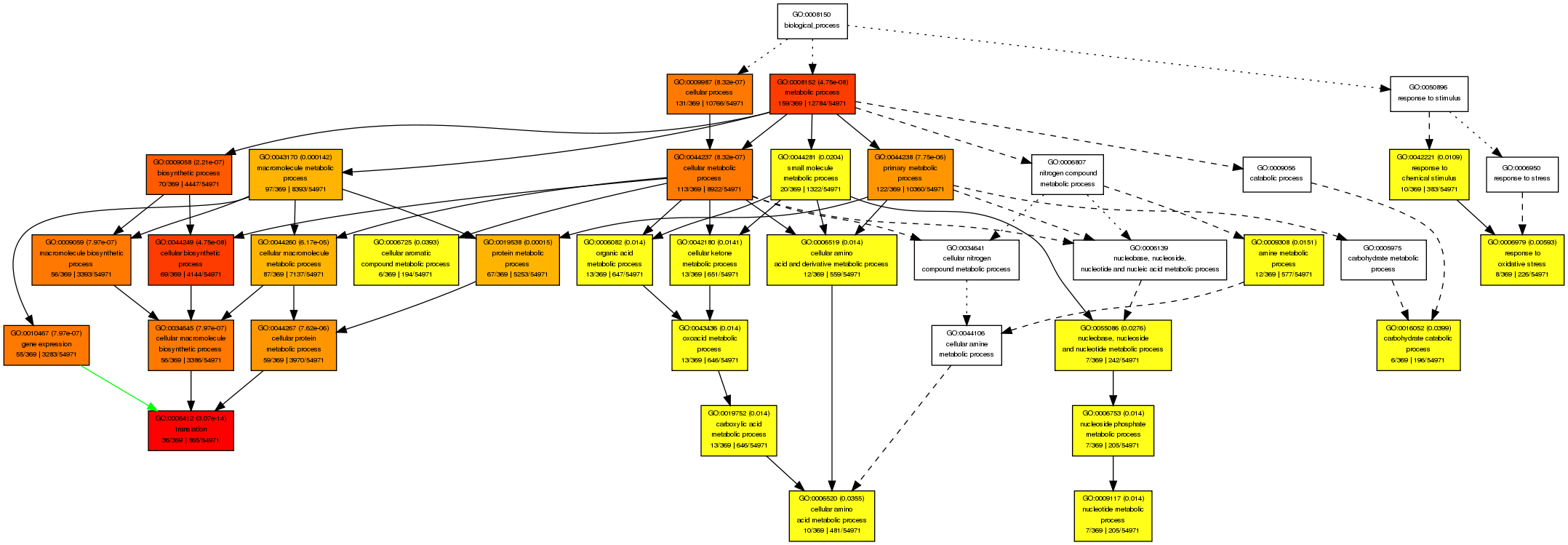

Supplement: File S8 — Singular enrichment analysis using AgriGO to identify enriched gene ontologies associated with buds of high tillering lines. (TIF) [file pone.0083772.s008.tif]
